# On-Demand Loco-Regional Treatment for Intrahepatic Lesions Improves Treatment Outcomes in Atezolizumab Plus Bevacizumab Therapy for Unresectable Hepatocellular Carcinoma

**DOI:** 10.3390/cancers18061021

**Published:** 2026-03-21

**Authors:** Kazuto Tajiri, Nozomu Muraishi, Eiki Ishizaka, Aiko Murayama, Yuka Hayashi, Ichiro Yasuda

**Affiliations:** The Third Department of Internal Medicine, Faculty of Medicine, University of Toyama, Toyama 930-0194, Japanyasudaic@med.u-toyama.ac.jp (I.Y.)

**Keywords:** atezolizumab, bevacizumab, locoregional treatment, combination therapy, objective response, neutrophil-lymphocyte ratio, intrahepatic control

## Abstract

Background/Objectives: Atezolizumab plus bevacizumab (Atez/Bev) is a standard treatment for unresectable hepatocellular carcinoma (HCC), but its anti-tumor efficacy remains limited. Combining intrahepatic locoregional treatment (IHLRT) with Atez/Bev has been explored as a strategy to overcome this limitation. This study aimed to clarify the significance of IHLRT in Atez/Bev treatment for unresectable HCC. Methods: Eighty consecutive patients with unresectable HCC treated with Atez/Bev were retrospectively analyzed. IHLRT was performed in patients with residual viable hepatic lesions amenable to locoregional treatment during Atez/Bev therapy. Anti-tumor response was evaluated by RECIST; and progression-free survival (PFS), overall survival (OS), potential biomarkers, and contributing factors to OS were also assessed. Results: IHLRT was selectively performed in 20 patients based on individual clinical conditions. Pretreatment characteristics were comparable between patients who did and did not receive IHLRT. Both best and initial tumor responses were superior in the IHLRT group, and PFS was significantly longer (16.2 vs. 8.4 months, *p* = 0.019), with comparable rates of severe treatment-related adverse events. On multivariate analysis, hepatic reserve function, objective response, neutrophil-to-lymphocyte ratio (NLR) and IHLRT were independent predictors of OS (HR: 2.17, 3.13, 0.58, and 1.62; *p* = 0.02, <0.01, 0.03 and 0.03, respectively). Although high NLR was a negative predictive factor, IHLRT appeared to mitigate the negative prognostic impact of an elevated NLR. Conclusions: On-demand, selective IHLRT during Atez/Bev treatment is well tolerated and provides superior and more durable tumor control, particularly in patients achieving an initial objective response.

## Simple Summary

Atezolizumab plus bevacizumab is a widely used drug combination for liver cancer that cannot be surgically removed, but it only achieves meaningful tumor shrinkage in around one-third of patients. To improve these outcomes, researchers have been investigating whether adding targeted, image-guided treatments directed at tumors within the liver can enhance the effectiveness of this drug combination. The underlying rationale and the best criteria for selecting patients for this combined approach, however, remain poorly understood. This study examined whether selectively applying such localized liver treatments during drug therapy improved patient outcomes. The findings suggest that this combined approach is safe, improves tumor control, and may extend survival, particularly in patients who show an early response to treatment. These results support a more individualized treatment strategy and lay the groundwork for ongoing clinical trials evaluating this approach.

## 1. Introduction

Hepatocellular carcinoma (HCC) is a life-threatening disease, ranking as the sixth most common cancer and the third leading cause of cancer-related death worldwide [1]. Most HCC patients are asymptomatic in the early stage, and about 70% of patients present with advanced disease, limiting the options for curative treatment such as resection or ablation [1]. Effective systemic chemotherapies are therefore essential to prolong survival following diagnosis. Atezolizumab plus bevacizumab (Atez/Bev), the first approved combination immunotherapy for HCC, has become a standard treatment for unresectable disease owing to its superior anti-tumor effects, survival benefits, and tolerable toxicity profile [2].

In patients with HCC and extrahepatic metastases, the primary cause of death has been reported to be hepatic failure attributable to intrahepatic tumor progression rather than extrahepatic disease [3,4]. Consequently, the importance of intrahepatic HCC control (IHC) has been increasingly recognized [4,5]. The significance of combining IHC with transcatheter arterial chemoembolization (TACE) was demonstrated in the LAUNCH trial, a recent phase 3 study of lenvatinib in advanced HCC patients, more than 50% of whom had extrahepatic metastases [6]. TACE is an established strategy for IHC; however, the post-TACE elevation of vascular endothelial growth factor (VEGF) is known to promote angiogenesis and subsequent tumor growth [7]. Synergistic effects between anti-angiogenic agents and TACE through normalization of tumor vasculature and improved drug delivery have also been reported [8]. Combining TACE with agents possessing anti-angiogenic potential may therefore represent a promising treatment strategy for unresectable HCC. Such benefits are particularly relevant to Atez/Bev, given that bevacizumab is a monoclonal antibody targeting VEGF. The effectiveness of combining Atez/Bev with TACE has recently been reported in real-world settings [9,10], and a prospective clinical trial evaluating this combination is currently underway [11].

Achieving a deep and durable anti-tumor response is a key determinant of prolonged overall survival (OS) with Atez/Bev, and early decline in tumor markers such as alpha-fetoprotein (AFP) is important for clinical decision-making [12]. In patients who demonstrate a remarkable response to Atez/Bev, the addition of IHC, so-called conversion therapy, may achieve complete response and allow treatment-free intervals [13]. The ability to predict objective response using pretreatment biomarkers is therefore highly desirable. Biomarkers reflecting the tumor microenvironment or hepatic reserve function, such as the neutrophil-to-lymphocyte ratio (NLR) and the albumin-bilirubin grade (ALBI), have been reported to predict therapeutic efficacy in both Atez/Bev [14,15] and combination therapy settings [16].

Despite growing interest in the role of IHC in unresectable HCC treated with Atez/Bev, its precise impact remains uncertain. In the present study, we performed a retrospective analysis to evaluate the significance of IHC in patients with unresectable HCC receiving Atez/Bev.

## 2. Materials and Methods

### 2.1. Patients

Patients with unresectable HCC and preserved hepatic function who started Atez/Bev treatment between October 2020 and July 2025 were retrospectively evaluated in the present study. HCC was diagnosed based on typical radiological and pathological findings and/or elevated levels of tumor markers such as alpha fetoprotein (AFP). Macrovascular invasion (MVI) and extrahepatic metastasis (EHM) were assessed in all patients by computed tomography (CT) and/or magnetic resonance imaging (MRI) before Atez/Bev treatment. Presence of anti-hepatitis C virus (HCV) antibody was defined as positive for HCV infection, and that of hepatitis B surface antigen for hepatitis B virus (HBV) infection. Patients without HCV or HBV infection, including those with alcoholic liver disease, nonalcoholic steatotic liver disease, or autoimmune liver disease, were classified as having ‘non-viral’ HCC. NLR, a marker of systemic inflammation, was also assessed. To evaluate hepatic reserve function, the albumin-bilirubin (ALBI) score and modified albumin-bilirubin (mALBI) grade were assessed in addition to Child–Pugh classification. Furthermore, HCC burden in the liver was evaluated using the ‘up-to-seven (UT7) criteria’ alongside Barcelona Clinic Liver Cancer (BCLC) staging. BCLC sub-classification was also performed based on BCLC staging and UT7 status [17]. Written informed consent was obtained from all participants before treatment, and the study was approved by our institutional ethics committee (Ethics Committee, University of Toyama, Approval Number: R2019131). This study was conducted in accordance with the Declaration of Helsinki.

### 2.2. Atez/Bev Treatment

Atezolizumab (Atez, anti PD-L1 antibody, 1200 mg) and bevacizumab (Bev, anti-vascular endothelial growth factor (VEGF) antibody, 15 mg/kg body weight) were administered intravenously every 3 weeks according to the manufacturer’s protocol. Atez/Bev treatment was continued until apparent tumor progression, defined as tumor growth of the whole target lesions, the appearance of multiple new lesions, the emergence of vessel invasion, or the occurrence of intolerable adverse events (AEs).

### 2.3. Combination of Locoregional Treatment

The addition of intrahepatic locoregional therapy (IHLRT) or locoregional therapy (LRT) for extrahepatic lesions was considered when intrahepatic lesions could not be controlled with Atez/Bev alone. For IHLRT, TACE was primarily considered for the control of residual intrahepatic lesions. Conventional-TACE (c-TACE) with miriplatin hydrate (Miripla^®^, Dainippon Sumitomo Pharma Co., Ltd., Tokyo, Japan) was used as the main chemo-embolic agent, with c-TACE using epirubicin hydrochloride (Epirubicin^®^, Nippon Kayaku Co., Ltd., Tokyo, Japan) as an alternative. In c-TACE procedures, a lipiodol emulsion containing the anti-cancer agent was injected into the super-selected tumor-feeding artery, followed by injection of 1 mm gelatin sponges (Gelpart^®^, Nippon Kayaku Co., Ltd., Tokyo, Japan). For lesions ≥ 5 cm diameter, drug-eluting bead (DEB)-TACE was performed using microspheres of 50–100 μm (Hepashere^®^, Nippon Kayaku Co., Ltd., Tokyo, Japan) loaded with epirubicin hydrochloride. DEB-TACE has been reported to be superior in inhibiting inflammatory responses and liver injury after TACE than conventional TACE [18]. In the present study, we prioritized maintaining hepatic reserve function after IHLRT so that Atez/Bev treatment could be continued. In the present study, embolization was not performed in two cases due to the absence of tumor staining. For lesions ≤ 2 cm diameter, radiofrequency ablation (RFA) was used as IHLRT. For complex lesions adjacent to or invading vessels, radiotherapy was considered. Selection of IHLRT was determined by the attending physician following multidisciplinary discussion. Atez/Bev treatment was discontinued 14–21 days before RFA or TACE and 7 days before radiotherapy. Following IHLRT, Atez/Bev treatment was either resumed or permanently discontinued depending on the individual case.

### 2.4. Tumor Response and Toxicity Assessment

Tumor response was evaluated by dynamic CT or MRI conducted every 9–12 weeks using the Response Evaluation Criteria in Solid Tumors (RECIST) during Atez/Bev treatment. Complete response (CR) was defined as disappearance of all target tumors; partial response (PR) as ≥30% decrease in the sum of the diameters of viable lesions; progressive disease (PD) as ≥20% increase in the sum; and stable disease (SD) as any case not meeting PR or PD criteria. In the present study, SD with <20% increase was further classified as progressive SD. The objective response rate (ORR) was defined as the proportion of patients achieving CR or PR, and disease control rate (DCR) as the proportion achieving CR, PR, or SD. Both initial and best tumor responses were recorded. The AFP ratio at 4 weeks after Atez/Bev initiation was also assessed, calculated as AFP at week 4 divided by pretreatment AFP. AEs occurring during Atez/Bev treatment were graded according to the National Cancer Institute Common Toxicity Criteria for Adverse Events (CTCAE) version 5.0. When unacceptable AEs related to Atez/Bev occurred, treatment was interrupted until symptoms resolved to grade 1 or lower as per CTCAE version 5.0.

### 2.5. Statistical Analysis

Categorical variables were compared by using chi-square or Fisher’s exact test, as appropriate, and continuous variables by the Mann–Whitney U test. Survival outcomes were analyzed using the Kaplan–Meier method and compared using log-rank tests. Factors associated with survival outcomes were assessed by multivariate analysis using the Cox proportional hazards model, while factors associated with ORR were assessed using logistic regression analysis. Receiver operating characteristic (ROC) curves and area under curve (AUC) were calculated to evaluate prediction of objective response. All statistical analyses were performed using SPSS version 19.0 (IBM, Armonk, NY, USA), with *p* < 0.05 deemed statistically significant.

## 3. Results

### 3.1. Characteristics of Patients Treated with Atez/Bev

This study included 80 patients treated with Atez/Bev. Their median age was 74 yr, and 65 (81.3%) were male (Table 1). Among them, 52 (65.0%) had non-viral etiology, 49 (61.3%) were at BCLC-C stage, 37 (46.3%) were MVI positive, and 22 (27.5%) were EHM positive. The majority of patients (72, 90.0%) were Child–Pugh grade A; however, 36 (45.0%) were classified as mALBI grade 2b or 3. ALBI score was similar between MVI present or not (mean ALBI, −2.11 vs. −2.30, *p* = 0.08, respectively). Atez/Bev was administered as first-line treatment in 63 patients (78.8%), with the remainder receiving it as second-line or later therapy. IHLRT was performed in 20 patients: TACE in 17, RFA in two, and radiotherapy in one. No significant differences in clinical characteristics were observed between patients with or without IHLRT (Table 1). The median number of Atez/Bev treatment cycles was eight (range, 1–30) with a median observation period of 13.8 (range, 1.0–55.3) months. IHLRT was initiated at a median of 7.3 (range, 3.7–35.1) months following initiation of Atez/Bev.

### 3.2. Anti-Tumor Effects and Survival Outcomes in Atez/Bev

**Figure 1:**
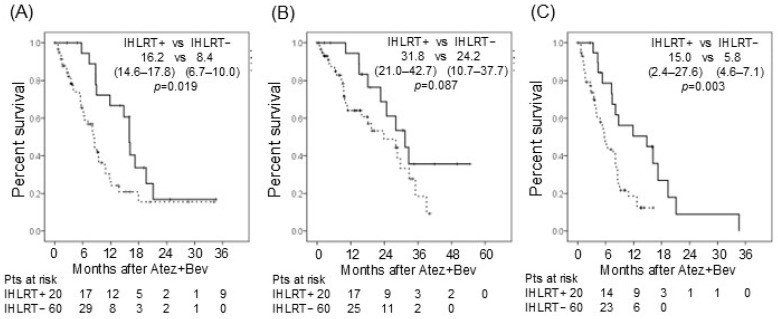
(**A**) Progression-free survival (PFS) after Atez/Bev administration in patients with and without IHLRT. Median PFS (95% confidence interval (CI)) is shown in the upper right of the panel. (**B**) Overall survival (OS) after Atez/Bev administration in patients with and without IHLRT. Median OS (95% CI) is shown in the upper right of the panel. (**C**) Treatment duration after Atez/Bev administration in patients with and without IHLRT. Median treatment duration (95% CI) is shown in the upper right of the panel. Solid line represents the survival curve with IHLRT+, whereas dotted line represents that with IHLRT−.

The initial and best objective response rates (ORR) according to RECIST were 20.8% and 39.0%, respectively (Table 2). CR was achieved in three patients in each group. In one IHLRT-treated patient, CR was achieved following the addition of RFA (Case 5, Supplementary Figure S1); in another, CR was achieved and maintained with the addition of radiotherapy (Case 9, Supplementary Figure S1); and in a third, CR was achieved after cessation of Atez/Bev (Case 6, Supplementary Figure S1). Patients who received IHLRT demonstrated significantly better disease control at both initial and best assessment (IHLRT+ vs. IHLRT-: initial ORR 50.0% vs. 15.7%; best ORR 75.0% vs. 36.8%) (Table 2). In the overall cohort, median PFS and OS were 9.5 months (95% CI: 7.2–11.8) and 28.5 months (95% CI: 22.3–34.7), respectively. PFS was significantly longer in the IHLRT+ group than in the IHLRT− group (16.2 vs. 8.4 months; 95% CI: 14.6–17.8 vs. 6.7–10.0; *p* = 0.019) (Figure 1A). OS also showed a trend toward improvement in the IHLRT+ group, although this did not reach statistical significance (31.8 vs. 24.2 months; 95% CI: 21.0–42.7 vs. 10.7–37.7; *p* = 0.087) (Figure 1B). Among the 20 patients who received IHLRT, six with progressive stable disease and six with stable disease were able to extend their treatment duration as a result of IHLRT (Supplementary Figure S1). Treatment duration was significantly longer in the IHLRT+ than the IHLRT− group (15.0 vs. 5.8 months; 95% CI: 2.4–27.6 vs. 4.6–7.1; *p* = 0.003) (Figure 1C).

### 3.3. Adverse Events

Treatment-related AEs are summarized in Table 3. No treatment-related deaths occurred. Severe TRAEs (grade ≥ 3) and immune-mediated adverse events (imAEs) requiring corticosteroids at a dose exceeding 20 mg were observed in three (15.0%) and two (10.0%) patients in the IHLRT+ group, respectively, rates comparable to those in the IHLRT− group. Of the two patients with imAEs, one developed interstitial nephritis after nine cycles of Atez/Bev and was treated with 40 mg of corticosteroid. Following recovery, Atez/Bev was resumed and continued for a total of 23 cycles, with TACE subsequently performed for intrahepatic progression after cycle 20. The second patient developed adrenal insufficiency after 10 cycles of Atez/Bev and was treated with 20 mg of corticosteroid; TACE was performed for intrahepatic progression after cycle 9. One patient with bile duct invasion developed grade 3 cholangitis after five cycles of Atez/Bev, during which Atez/Bev was withheld and TACE was performed. Liver injury and fever, both grade 2 or lower, were observed more frequently in the IHLRT+ group but were manageable in all cases.

### 3.4. Factors Contributing to OS

**Figure 2:**
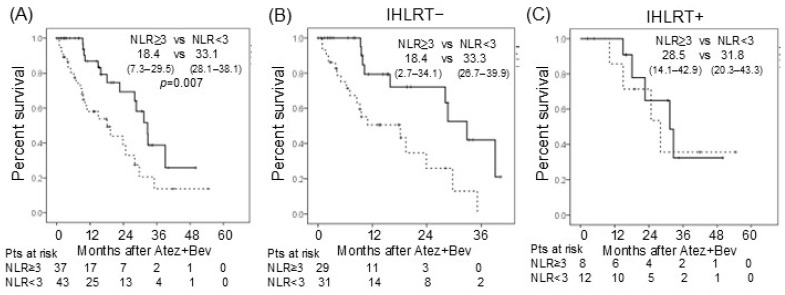
(**A**) Overall survival (OS) after Atez/Bev administration according to NLR in the entire cohort. Median PFS (95% confidence interval (CI)) is shown in the upper right panel. (**B**) Overall survival (OS) after Atez/Bev administration according to NLR in patients without IHLRT. Median OS (95% CI) is shown in the upper right panel. (**C**) Overall survival (OS) after Atez/Bev administration according to NLR in patients with IHLRT. Median treatment duration (95% CI) is shown in the upper right panel. Solid line represents the survival curve with NLR ≥ 3, whereas dotted line represents that with NLR < 3.

On univariate analysis, preserved mALBI grade, objective response, NLR, and IHLRT were each significantly associated with favorable OS (Table 4). BCLC stage and treatment line did not contribute significantly to OS in this cohort. Multivariate analysis identified objective response as the strongest independent predictor of OS (HR: 3.13, *p* < 0.01), followed by preserved mALBI grade (HR: 2.17), NLR (HR: 0.58) and IHLRT (HR: 1.62), both of which were also independently significant. Among pretreatment biomarkers previously reported to predict objective response, NLR demonstrated the highest predictive value in this cohort (AUC: NLR 0.364, ALBI 0.331, AFP ratio at 4 weeks 0.301, AFP 0.272) (Supplementary Figure S2). Patients with NLR < 3 had significantly better OS than those with NLR ≥ 3 in the overall cohort (Figure 2A) and in the IHLRT− subgroup (Figure 2B). However, in the IHLRT+ subgroup, OS was similar regardless of NLR status (Figure 2C), suggesting that IHLRT may mitigate the adverse prognostic impact of an elevated NLR.

## 4. Discussion

In the present study, the addition of on-demand IHLRT during Atez/Bev treatment in patients with advanced HCC improved treatment outcomes by enhancing anti-tumor response and prolonging the tumor-controlled state. Achieving and sustaining anti-tumor effects are essential for prolonged survival during Atez/Bev treatment, and IHLRT can meaningfully contribute to this goal. Although elevated NLR is a potential pretreatment biomarker for predicting anti-tumor response, IHLRT may be able to overcome its negative prognostic impact.

Objective anti-tumor response is a key determinant of long-term survival with Atez/Bev, and both the depth and duration of response are associated with better outcomes [12,19,20]. Achieving CR through IHLRT, so-called curative conversion, can result in a treatment-free state, which is regarded as a therapeutic goal in patients with unresectable HCC [13]. Patients who demonstrate a good response during Atez/Bev may therefore be well-suited candidates for the addition of IHLRT. Hosui et al. reported the benefit of adding IHLRT in patients showing stable disease during Atez/Bev, with a survival advantage observed in the Atez/Bev group but not in those receiving lenvatinib, suggesting that immune modulation may play a role in the benefit of additive IHLRT [21]. Su et al. further reported that combining IHLRT with sequential systemic therapy after progression on Atez/Bev provided better tumor responses and survival benefits than systemic therapy alone, implying that IHLRT may also modulate the tumor microenvironment beyond its direct anti-tumor effects [22]. Nakabori et al. demonstrated the effectiveness of on-demand, selective IHLRT in both CR-oriented and progression-salvage settings during Atez/Bev [9]. Taken together, the benefit of additive IHLRT during Atez/Bev treatment may be independent of treatment response, potentially mediated through modification of the tumor microenvironment. Our data showed that an initial anti-tumor response was more frequently observed in patients who received IHLRT, suggesting that the benefit of IHLRT may be optimized when overall intrahepatic disease is adequately controlled. In a previous report, progression-salvage IHLRT was applied selectively to treat solitary or limited drug-resistant lesions [9]. The role of progression-salvage IHLRT within Atez/Bev treatment warrants further investigation. Furthermore, TACE was the most frequent modality as IHLRT in present study. The technique or device of TACE, especially embolic reagents, has evolved to improve treatment outcomes [23,24]. Optimization of IHLRT should be considered continuously.

Predicting objective anti-tumor response remains an important unmet need in Atez/Bev treatment. Although no established biomarkers exist, pretreatment hepatic reserve function, systemic inflammatory markers, and their combinations are considered promising predictive factors in HCC patients receiving Atez/Bev [25,26]. Early AFP response has also been shown to be a valuable predictive marker [27,28]. In the present study, NLR predicted objective response more effectively than ALBI or AFP response, although none reached acceptable discriminatory performance as a standalone predictor (AUC < 0.5 for all) (Supplementary Figure S2). NLR is a recognized biomarker reflecting systemic inflammation and the inflammatory tumor microenvironment across multiple cancers, including HCC [29]. Consistent with this, OS differed significantly according to NLR in the present cohort (Figure 2A). Interestingly, this survival difference was abolished in patients who received IHLRT (Figure 2B,C), suggesting that IHLRT may help patients with elevated NLR achieve and maintain objective anti-tumor responses. Whether this effect is attributable to direct treatment of Atez/Bev-resistant lesions, abscopal effects, or other mechanisms remains to be clarified in future studies. In addition to NLR, the effectiveness of other inflammation-based biomarkers such as lymhocyte-to-monocyte ratio (LMR) or platelet-to-lymphocyte ratio has been suggested in HCC or secondary liver cancers [30,31,32,33]. A recent study showed LMR is a prognostic biomarker in patients also treated with durvalumab plus tremelimumab [34]. LMR has been suggested as a biomarker reflecting the balance between immune response and tumor-promoting inflammation [35]. Selection and optimization of these biomarkers should be investigated in future studies.

The present study has several limitations. First, its retrospective design and limited sample size are the primary constraints, potentially reducing statistical power. The retrospective nature might lead to selection bias or some unrecognized bias. Prospective studies with larger samples are desirable. Furthermore, all treatment decisions were made by individual physicians within the Japanese public healthcare system at a single center, which may limit the generalizability of these findings to other patients, institutions, or healthcare settings. However, the combination of IHLRT showed improved tumor control and survival outcomes with manageable toxicity, and may benefit patients with an unfavorable tumor microenvironment, which is a clinically meaningful finding given the limited anti-tumor activity of Atez/Bev alone. Second, the optimal timing and selection criteria for IHLRT remain uncertain. In the present study, IHLRT was selected with the aim of achieving reliable and safe local tumor control, and no deterioration of hepatic reserve function following IHLRT was observed. Preservation of liver function is a critical consideration throughout HCC management, and on-demand, selective IHLRT appears to be a viable approach in this context. Third, only a limited set of single biomarkers was evaluated. The prognostic and predictive significance of biomarkers in Atez/Bev for unresectable HCC continues to be an area of active investigation. Recent work has highlighted the potential of machine learning-based ensemble biomarker models over individual or simple biomarkers in advanced HCC treated with Atez/Bev [25], and future studies exploring multi-marker approaches are warranted.

## 5. Conclusions

On-demand, selective IHLRT in combination with Atez/Bev is well tolerated and can achieve better and more durable tumor control, particularly in patients demonstrating a response to Atez/Bev. Although the precise mechanism underlying the benefit of IHLRT combination remains unclear, it may also confer a survival advantage in patients with elevated NLR.

## Figures and Tables

**Table 1:** Characteristics of HCC patients treated with Atez/Bev.

	Total	IHLRT+	IHLRT−	*p*
Age (years)	74 (37–90)	73.5 (39–87)	74.5 (37–90)	0.59
Sex (M/F)	65/15	16/4	49/11	0.87
HBV/HCV/Non-Viral	13/15/52	4/3/13	9/12/39	0.63
PS (0, 1/2)	76/4	20/0	56/4	0.18
BCLC stage (B/C)	31/49	8/12	23/37	0.90
MVI (present)	37	11	26	0.37
EHM (present)	22	5	17	0.78
Child–Pugh (A/B)	72/8	19/1	53/7	0.73
mALBI (1, 2a/2b, 3)	44/36	15/5	29/31	0.15
Max IHL size (cm)	4 (0–20)	6.5 (1–16)	4 (0–20)	0.25
IHL number	3 (0–>20)	2.5 (1–10)	3 (0–>20)	0.30
NLR	2.9 (0.8–22.4)	2.6 (0.9–4.6)	2.9 (0.8–22.4)	0.40
AFP (ng/mL)	13.0 (1.7–202,111)	11.5 (1.7–202,111)	22.1 (2.2–14,334)	0.94
DCP (AU/L)	344 (9–162,964)	669 (15–15,403)	221 (9–162,964)	0.66
Treatment line (F/L)	63/17	16/4	47/13	0.88

Median (range) or *N*/*N*, number of cases is shown. HCC, hepatocellular carcinoma; Atez + Bev, atezolizumab plus bevacizumab; IHLRT, intrahepatic locoregional therapy; M, male; F, female; HBV, hepatitis B virus; HCV, hepatitis C virus; PS, performance status; BCLC, Barcelona Clinic Liver Cancer; MVI, major vessel invasion; EHM, extrahepatic metastasis; mALBI, modified albumin-bilirubin; IHL, intrahepatic lesion; NLR, neutrophil-to-lymphocyte ratio; AFP, alpha fetoprotein; DCP, des-gamma-carboxythrombin; F, first line; L, later line.

**Table 2:** Anti-tumor response in Atez/Bev treatment.

	All	IHLRT+ (n = 20)	IHLRT− (n = 57)	*p*
	Initial (%)/Best (%)	Initial (%)/Best (%)	Initial (%)/Best (%)	Initial/Best
CR	0 (0)/3 (3.9)	0 (0)/2 (10.0)	0 (0)/3 (5.3)	1.00/0.60
PR	16 (20.8)/27 (35.1)	10 (50.0)/13 (65.0)	9 (15.7)/18 (31.6)	0.005/0.02
SD	54 (70.1)/40 (51.9)	10 (50.0)/5 (25.0)	41 (71.9)/29 (50.9)	0.10/0.07
PD	7 (9.1)/7 (9.1)	0 (0)/0 (0)	7 (12.3)/7 (12.3)	0.18/0.18
ORR	16 (20.8)/30 (39.0)	10 (50.0)/15 (75.0)	9 (15.7)/21 (36.8)	0.005/0.004
DCR	70 (90.9)/70 (90.9)	20 (100)/20 (100)	50 (87.7)/50 (87.7)	0.18/0.18

Proportions (%) of cases among 77 cases with anti-tumor response evaluated by RECIST. Anti-tumor response of 3 cases with IHLRT− were not evaluated. IHLRT, intrahepatic locoregional therapy; CR, complete response; PR, partial response; SD, stable disease; PD, progressive disease; ORR, objective response rate; DCR, disease control rate.

**Table 3:** Treatment-related adverse events in Atez/Bev treatment.

	IHLRT+ (n = 20)	IHLRT− (n = 60)	*p*
Treatment-related AEs (≥Grade 3)	3 (15.0)	8 (13.3)	1.00
Immune-mediated AEs	3 (15.0)	8 (13.3)	1.00
required ≥20 mg steroid	2 (10.0)	5 (8.3)	1.00
Hypertension	6 (30.0)	14 (23.3)	0.56
Proteinuria	5 (25.0)	11 (18.3)	0.53
Hypothyroidism	2 (10.0)	5 (8.3)	1.00
Skin rush	1 (5.0)	2 (6.7)	1.00
Diarrhea	1 (5.0)	2 (6.7)	1.00
Liver injury	6 (30.0)	5 (8.3)	0.02
Fatigue	1 (5.0)	2 (6.7)	1.00
Fever	4 (20.0)	2 (6.7)	0.03

Number of cases (percent) with evaluation of anti-tumor response by CTCAE v4.0 is shown. HLRT, intrahepatic locoregional therapy; AEs, adverse events.

**Table 4:** Factors predicting OS in HCC patients treated with Atez/Bev.

	Univariate			Multivariate		
	HR	95% CI	*p*	HR	95% CI	*p*
mALBI (1, 2a)	2.76	1.50–5.07	<0.01	2.17	1.13–4.17	0.02
BCLC (C)	0.76	0.43–1.35	0.35	0.85	0.46–1.55	0.59
Objective response (CR, PR)	2.81	1.51–5.21	<0.01	3.13	1.60–6.12	<0.01
Treatment line (First)	0.61	0.33–1.14	0.12	0.74	0.39–1.40	0.35
NLR (≥3)	0.46	0.21–0.80	<0.01	0.58	0.26–0.90	0.03
IHLRT (+)	2.14	1.11–4.10	0.02	1.62	1.09–4.16	0.03

OS, overall survival; HCC, hepatocellular carcinoma; Atez + Bev, atezolizumab plus bevacizumab; HR, hazard ratio; CI, confidence interval; mALBI, modified albumin-bilirubin; BCLC, Barcelona Clinic Liver Cancer; CR, complete response; PR, partial response; IHLRT, intrahepatic locoregional.

## Data Availability

All data on which the conclusions of this study relied are shown in the manuscript. Details are available from the corresponding author upon reasonable request.

## Supplementary Materials

The following supporting information can be downloaded at https://www.mdpi.com/article/10.3390/cancers18061021/s1, Figure S1: Clinical course in each patient treated with Atez/Bev with IHLRT; Figure S2: Receiver operating characteristic (ROC) curve to predict objective response with each parameter.
