# Supplementary material for: On-Demand Loco-Regional Treatment for Intrahepatic Lesions Improves Treatment Outcomes in Atezolizumab Plus Bevacizumab Therapy for Unresectable Hepatocellular Carcinoma
Source: Cancers (Basel). 2026 Mar 21;18(6):1021. doi: 10.3390/cancers18061021 (PMC13025880; doi:10.3390/cancers18061021)
Supplement: Supplementary file 1 [file cancers-18-01021-s001.zip › cancers-4203022-supplementary.pdf]

cancers

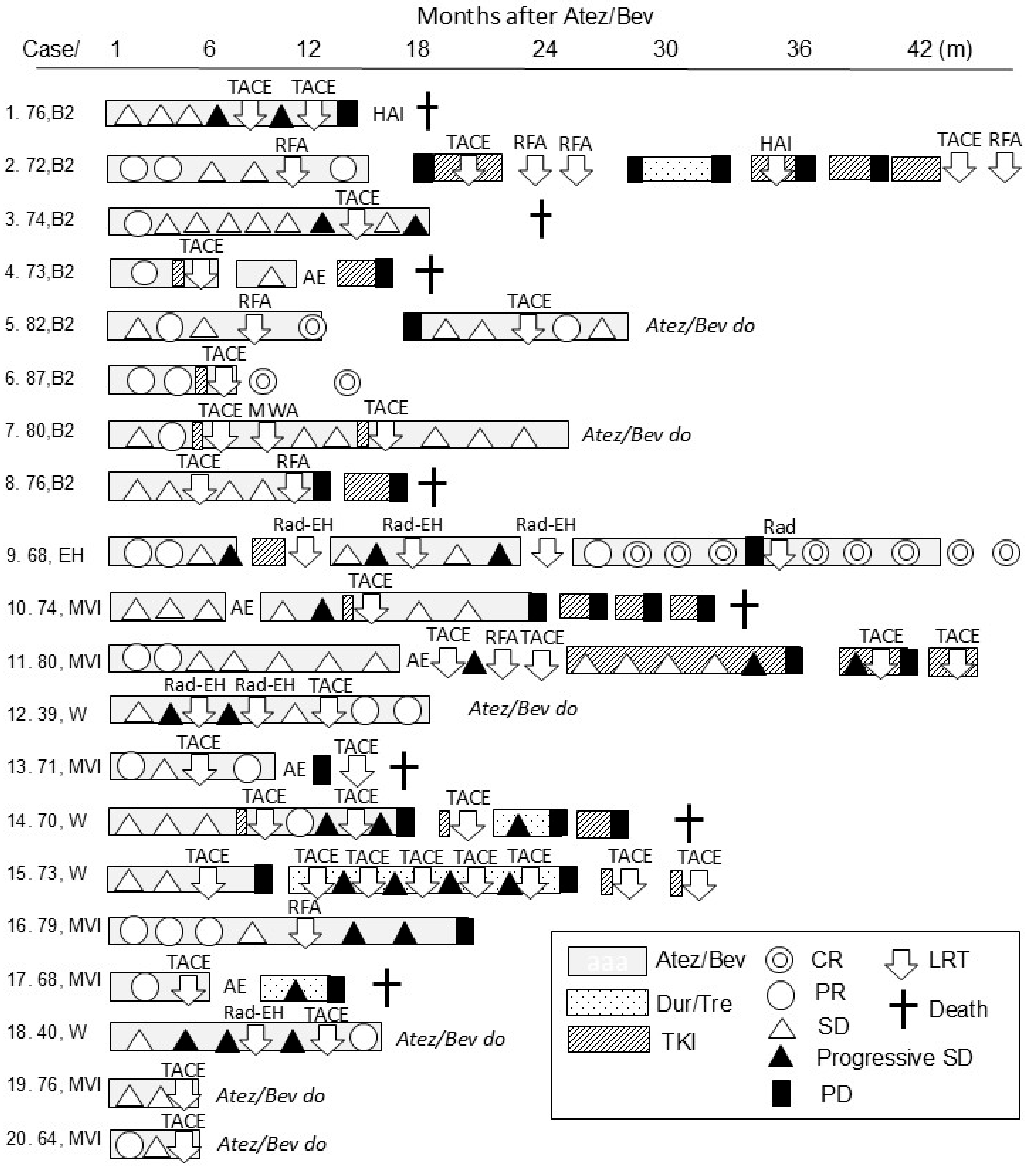

Figure S1. Clinical course in each patient treated with Atez/Bev with IHRT. Each line indicates clinical course of each case. At the left end of each line, age and BCC staging are shown. B2, BCLC-B2; EH, extrahepatic metastasis; MV, major vessel invasion; W, both EH and MVI.

 https://doi.org/10.3390/cancers18061021

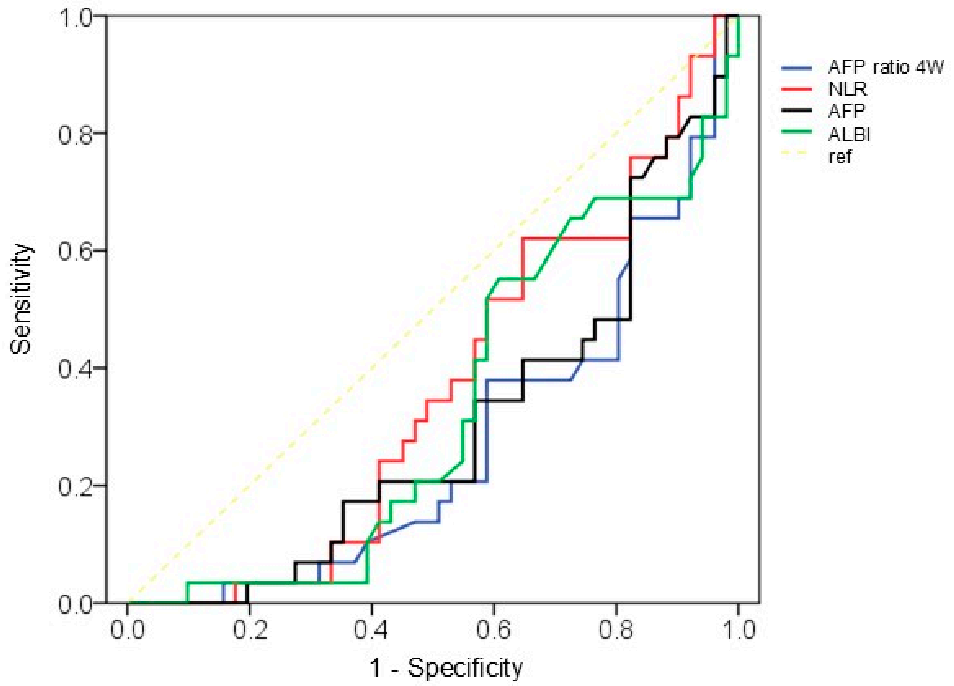

Figure S2. Receiver operating characteristic (ROC) curve to predict objective response with each parameter.

Disclaimer/Publisher's Note: The statements, opinions and data contained in all publications are solely those of the individual author(s) and contributor(s) and not of MDPI and/or the editor(s). MDPI and/or the editor(s) disclaim responsibility for any injury to people or property resulting from any ideas, methods, instructions or products referred to in the content.
